# Developmental Characterization of Schizophrenia-Associated Gene Zswim6 in Mouse Forebrain

**DOI:** 10.3389/fnana.2021.669631

**Published:** 2021-05-13

**Authors:** Chuan-Chie Chang, Hsiao-Ying Kuo, Shih-Yun Chen, Wan-Ting Lin, Kuan-Ming Lu, Tetsuichiro Saito, Fu-Chin Liu

**Affiliations:** ^1^Institute of Neuroscience, National Yang Ming Chiao Tung University, Taipei, Taiwan; ^2^Department of Biological Science and Technology, National Yang Ming Chiao Tung University, Taipei, Taiwan; ^3^Department of Developmental Biology, Graduate School of Medicine, Chiba University, Chiba, Japan

**Keywords:** basal ganglia, striatum, schizophrenia, dopamine, Zswim

## Abstract

Schizophrenia is a devastating neuropsychiatric disease with a globally 1% life-long prevalence. Clinical studies have linked *Zswim6* mutations to developmental and neurological diseases, including schizophrenia. *Zswim6*’s function remains largely unknown. Given the involvement of *Zswim6* in schizophrenia and schizophrenia as a neurodevelopmental disease, it is important to understand the spatiotemporal expression pattern of *Zswim6* in the developing brain. Here, we performed a comprehensive analysis of the spatiotemporal expression pattern of *Zswim6* in the mouse forebrain by *in situ* hybridization with radioactive and non-radioactive-labeled riboprobes. *Zswim6* mRNA was detected as early as E11.5 in the ventral forebrain. At E11.5–E13.5, *Zswim6* was highly expressed in the lateral ganglionic eminence (LGE). The LGE consisted of two progenitor populations. Dlx^+^;Er81^+^ cells in dorsal LGE comprised progenitors of olfactory bulb interneurons, whereas Dlx^+^;Isl1^+^ progenitors in ventral LGE gave rise to striatal projection neurons. *Zswim6* was not colocalized with Er81 in the dorsal LGE. In the ventral LGE, *Zswim6* was colocalized with striatal progenitor marker *Nolz-1*. *Zswim6* was highly expressed in the subventricular zone (SVZ) of LGE in which progenitors undergo the transition from proliferation to differentiation. Double labeling showed that *Zswim6* was not colocalized with proliferation marker Ki67 but was colocalized with differentiation marker Tuj1 in the SVZ, suggesting *Zswim6* expression in early differentiating neurons. *Zswim6* was also expressed in the adjacent structures of medial and caudal ganglionic eminences (MGE, CGE) that contained progenitors of cortical interneurons. At E15.5 and E17.5, *Zswim6* was expressed in several key brain regions that were involved in the pathogenesis of schizophrenia, including the striatum, cerebral cortex, hippocampus, and medial habenular nucleus. *Zswim6* was persistently expressed in the postnatal brain. Cell type analysis indicated that *Zswim6* mRNA was colocalized with *D1R*-expressing striatonigral and *D2R*-expressing striatopallidal neurons of the adult striatum with a higher colocalization in striatopallidal neurons. These findings are of particular interest as striatal dopamine D2 receptors are known to be involved in the pathophysiology of schizophrenia. In summary, the comprehensive analysis provides an anatomical framework for the study of *Zswim6 function and Zswim6-associated neurological disorders*.

## Introduction

The members of *ZSWIM* family contain a SWIM domain of CxCxnCxH motif of predicted zinc chelating residues that may be involved in DNA binding and protein–protein interaction (Makarova et al., 2002). *ZSWIM* gene family consists of 10 members, *MAP3K1* and *ZSWIM1* to *ZSWIM9* (Makarova et al., 2002). Of the *Zswim* family members, *Zswim6* is the member that is expressed at a high level in the adult mouse brain (Lein et al., 2007). A previous study of *Zswim6* knockout mice indicates that *Zswim6* regulates the morphology of striatal neurons and motor function (Tischfield et al., 2017). In addition to the striatum, *Zswim6* is also expressed in the amygdala. NMDA-induced downregulation of *Zswim6* was observed in the central nucleus of amygdala of *Fyn* knockout mice (Kai et al., 2006).

The biological importance of *Zswim6* has been implicated in studies of diseases. Clinical studies have shown that patients with *Zswim6* genetic mutations are associated with developmental and neurological diseases, including schizophrenia, acromelic frontonasal dysostosis and intellectual disability (Ripke et al., 2013; Lencz et al., 2014; Schizophrenia Working Group of the Psychiatric Genomics Consortium, 2014; Smith et al., 2014; Twigg et al., 2016; Palmer et al., 2017). Interestingly, *ZSWIM6* is ranked in the top five schizophrenia-associated genes that are linked to the MAPK signaling pathway (Pers et al., 2016). An animal model study has shown that Zswim6 is downregulated in the amygdala of *MECP2* duplicated mice, a mouse model of *MECP2* duplication syndrome (Samaco et al., 2012).

We have previously characterized the expression pattern of *Zswim5*, a paralogue of *Zswim6*, in the developing mouse forebrain (Chang et al., 2020). In the present study, we characterize the developmental expression pattern of *Zswim6* mRNA in the mouse forebrain. Unlike *Zswim5* that is enriched in the medial ganglionic eminence (MGE) of the ventral forebrain where progenitors of cortical and striatal interneurons reside (Marin et al., 2000), *Zswim6* is highly expressed in the adjacent structure of lateral ganglionic eminence (LGE) where progenitors of striatal projection neurons are located (Olsson et al., 1995), which is in agreement with previous studies (Lein et al., 2007; Chang, 2009; Tischfield et al., 2017; Mayer et al., 2018). Concerning temporal regulation, in contrast to the downregulation of *Zswim5* in the postnatal brain, *Zswim6* expression is persistently expressed in the postnatal forebrain. Therefore, two paralogues of *Zswim5* and *Zswim6* have distinct spatiotemporal expression patterns in the developing mouse forebrain, which may endow them with different neurobiological functions.

## Materials and Methods

### Animals and Sample Collection

ICR mice (BioLASCO Inc., Taiwan) were used in this study. All the experimental mice were bred in the Animal Center of National Yang Ming Chiao Tung University, and the experimental methods were approved by the Institutional Animal Care and Use Committee. To mark the developmental stages of mice, noon of the day observed vaginal plug in the pregnant dam was defined as embryonic day 0.5 (E0.5) of embryos. The day pups were delivered, was defined as postnatal day 0 (P0). The brains of ICR mice at E11.5, E12.5, E13.5, E15.5, E17.5, P0, P7, P14–15, and adults were harvested with the following protocols. Time-pregnant dams were deeply anesthetized with i.p. injection of sodium pentobarbital, and the heads of embryos were immediately fixed with 4% paraformaldehyde (PFA) in 0.1 M phosphate-buffered saline (PBS, pH 7.4) at 4°C overnight. For collecting postnatal brain, mice were perfused with 0.9% NaCl followed by 4% PFA/PBS transcardially. After postfixation overnight, brains were cryoprotected by 30% sucrose in PBS at 4°C for two nights. All brains were immediately frozen with dry ice and then stored at −70°C before cryostat sectioning. For cryo-sectioning, frozen brains were embedded in the Cryo-Gel (Instrumedics) and were instantly frozen with dry ice. The brains were sectioned into 20 μm or 25 μm with a cryostat (Leica CM1900). After sectioning, the slides were air-dried in a desiccator connected with a vacuum pump for a few hours and stored at −20°C.

### Subcloning of *Zswim6* Probes for *In situ* Hybridization

*Zswim6* cDNA fragment with a truncated N-terminal subcloned within pBC SK+ vector (mKIAA1577) was kindly provided by the Kazusa organization in Japan. The truncated *Zswim6* cDNA fragment (4,218 bp) was inserted into multiple cloning sites of the Chloramphenicol resistance pBC SK+ vector (3,400 bp) between the XhoI and NotI site. The mKIAA1577 was first digested with restriction enzyme SpeI (NEB) at 37°C overnight, generating two fragments long 1,245 bp and 6,375 bp. The 6,375 bp DNA fragment was then eluted out by elution kit (Geneaid) and digested with NotI (NEB) at 37°C overnight. After enzyme digestion, the 1,331 bp target fragment originally located between the SpeI site (NEB) at 2,888 bp and NotI site (NEB) at 4,218 bp of the mKIAA1577 sequence was generated. In the meantime, pBluescript SK (−) was also digested to form the 2,983 bp targeting vector by double enzyme digestion using SpeI (NEB) and NotI (NEB) at 37°C overnight. The 1,331 bp fragment of mKIAA1577 was then ligated with the 2,983 bp pBluescript SK (−) targeting vector by T4 ligase (NEB) at 16°C overnight. The ligation products were transformed using DH5α (Yeastern Biotech) and selected by antibiotics. Clones with correct ligation were identified by PCR using T3 and T7 primers with the following PCR condition: 94°C for 3 min, 30 cycles of denaturation (94°C for 30 s, annealing (55°C for 30 s, and extension (72°C for 2 min), 72°C for 2 min, and finally stopped at 4°C. The positive clones were further checked with restriction enzymes and PstI (NEB) and EcoRV (NEB) to generate fragments of 1,273 bp; 3,058 bp; 275 bp and 4,056 bp respectively. Another *Zswim6* riboprobe was generated by PCR cloning according to the previous study (Tischfield et al., 2017).

### Synthesis of Digoxigenin (Dig)-Labeled Riboprobes

The *Zswim6* riboprobes were generated as described above. The rat Drd1 and Drd2L riboprobes were kindly provided by K. Kobayashi at Fukushima Medical University, Fukushima, Japan. The riboprobes were synthesized by *in vitro* transcription with digoxigenin (dig) or fluorescein (FITC) RNA labeling mix (Roche). Briefly, respective linearized template plasmid was mixed with 5× transcription buffer, 0.1 M dithiothreitol (DTT), Dig-labeling mix (Roche), RNasin (Promega), respective RNA polymerase (T3, T7, SP6, Promega), and DEPC-treated H_2_O at 37°C for 2 h according to manufacturer’s instruction (Promega). The DNA template was digested with DNase RQ1 at 37°C for 30 min. After stopping polymerase reaction by adding 0.2 M EDTA (pH 8.0) and placed on ice for 5 min, STE buffer (0.1 M NaCl, 10 mM Tris-HCl, pH 8.0; 1 mM EDTA, pH 8.0) and 3 μl 1 M DTT were added. Finally, the probes were further purified with G-50 mini Quick Spin Columns (Roche). The size and quality of probes had been checked by gel electrophoresis before DNase RQ1 treatment and after purification.

### Digoxigenin-Labeled *In situ* Hybridization

Slides stored at −20°C were first air-dried at room temperature (RT) for 10 min and then further dried in the desiccator connected with a vacuum pump. After stopping polymerase reaction by adding 0.2 M EDTA (pH 8.0) and being placed on ice for 5 min, STE buffer (0.1 M NaCl, 10 mM Tris-HCl, pH 8.0; 1 mM EDTA, pH 8.0) and 3 μl 1 M DTT were added. Finally, the probes were further purified with G-50 mini Quick Spin Columns (Roche). The size and quality of probes had been checked by gel electrophoresis before DNase RQ1 treatment and after purification for more than 1 h. Embryonic sections were washed in 0.01 M PBS for 5 min and treated with 0.1% Triton X-100 in 0.01 M PBS for 5 min. Postnatal sections were post-fixed in 4% PFA/PBS for 30 min on ice and then treated with 0.3% Triton X-100 in 0.01 M PBS for 15 min. After washing with 0.01 M PBS, all sections were incubated in 0.2 N HCl in DEPC-treated H_2_O for 20 min. Sections were treated with proteinase K (PK, 10 μg/ml, MDBio) in 0.01 M PBS at 37°C for 2–5 min. After washing in 0.01 M PBS, sections were fixed with 4% PFA/0.01 M PBS for 5 min and treated with glycine (2 μg/ml) in 0.01 M PBS for 15 min twice. Sections were then prehybridized with 50% deionized formamide (Sigma) in 2× standard saline citrate (SSC, 300 mM NaCl, 30 mM sodium citrate, pH 7.0) at 65°C for 90 min in a humid oven. Diluted probes ranging from 1:250 to 1:1,000 in the hybridization solution (50% formamide; 10% dextran sulfate; 0.3 M NaCl; 0.01 M Tris, pH 8.0; 500 μg/ml yeast tRNA; 10 mM DTT; 1 mM EDTA, pH 8.0, and 1× Denhardt’s solution) were denatured at 90°C for 10 min, and applied onto the samples. After hybridization at 65°C for 16 h, sections were washed with 5× SSC for 5 min and then incubated in 50% formamide (Sigma)/2× SSC for 1 h. Before and after treating with RNase A (20 μg/ml) at 37°C for 30 min, sections were incubated in 10 mM Tris-HCl (pH 8.0) and 500 mM NaCl for 10 min, respectively. The sections were then washed with 2× SSC once, 0.2× SSC twice for 20 min at 65°C and TNT buffer (150 mM NaCl, 100 mM Tris pH 7.5) for 10 min sequentially. After blocking the sections [2% blocking reagent (Roche) and 20% sheep serum in TNT buffer] for 60 min, alkaline phosphatase (AP)-conjugated sheep anti-digoxigenin antibody (1:1,000, Roche, RRID:AB_514497) were incubated for 90 min. After washing the sections in TNT buffer and alkaline buffer (100 mM Tris-HCl pH 9.5, 100 mM NaCl), signals were detected by colorimetric procedure with 5-Bromo-4-chloro-3-indolyl phosphate (BCIP, Roche) and Nitro blue tetrazolium chloride (NBT, Roche) in the alkaline buffer. For the fluorescent *in situ* hybridization, all TNT buffer contained an additional 0.1% Tween-20. Sections were washed with 0.1% H_2_O_2_ in TNT. After the blocking procedures described above, horseradish peroxidase (HRP)-conjugated sheep anti-digoxigenin (1:100, Roche; RRID:AB_514500) were incubated overnight. On the next day, the signals were detected by Cy3 conjugated tyramide [1:1,000 in dilution buffer, tyramide signal amplification (TSA, PerkinElmer)] for 10 min. The fluorescent signals were analyzed with fluorescence microscopy (Eclipse E800M, Nikon; BX51, Olympus) or confocal microscopy (SP2, Leica; LSM880, Zeiss).

### Dual *In situ* Hybridization

Following *in situ* hybridization protocols described above, sheep anti-FITC antibody (1:100, Roche, RRID:AB_840257) was used to identify the first type of transcript. After detection of FITC signals, the HRP activity for detecting the first antibody was bleached by 0.1% H_2_O_2_ in TNT buffer for 15 min. Then the sections were again incubated in 2% blocking solution [2% blocking reagent (Roche), 20% sheep serum in the TNT buffer] for an additional 60 min. Finally, sheep anti-Dig antibody (1:100, Roche, RRID:AB_514500) was used to detect the second transcript target with overnight incubation. On the next day, the TSA system was applied with a different fluorescence according to the manufacturer’s instruction.

### Radioactive *In situ* Hybridization

After air-drying in the desiccators for over 60 min, the sections were post-fixed with 10% formaldehyde in potassium PBS (KPBS, 1.5 M NaCl, 0.03 M KH_2_PO_4_, and 0.2 M K_2_HPO_4_) and followed by treatment with 10 μg/ml PK in buffer containing 0.1 M Tris (pH 8.0), and 0.05 M EDTA (pH 8.0) for 20 min at 37°C. After rinsing in DEPC-treated H_2_O for 3 min, the sections were incubated for 10 min in 0.1 M Triethanolamine solution containing 0.25% acetic anhydride (TEA, pH 8.0), and were then washed by 2× SSC buffer. After dehydration with successive rinses of ethanol (50%, 70%, 95%, and 100% twice, each for 3 min), the sections were air-dried for 2 h and further hybridized with ^35^S-labeled antisense probes for 16 h at 58°C. The probe was mixed with the hybridization solution (10^7^ counts/min/ml). Before applying the hybridization mixture onto the sections, the probes were denatured for 5 min at 65°C. On the next day, the sections were first washed with 4× SSC 7 min four times and were treated with 10 μg/ml RNase A in buffer containing 0.5 M NaCl, 10 mM Tris (pH 8.0), and 1 mM EDTA (pH 8.0) for 30 min at 37°C. The sections were then washed with 2× SSC twice, 1× SSC and 0.5× SSC once (each for 5 min), 0.1× SSC for 30 min at 50°C, and 0.1× SSC for another 5 min. All the SSC washing buffers used above contained 1 mM DTT. After dehydration with successive 5-min rinses in ethanol (50%, 70%, 95%, and 100% twice, each for 5 min) and drying in the desiccators, the sections were exposed to X-ray films for detecting ^35^S-labeled signals by autoradiography.

### Immunohistochemistry

For dual *in situ* hybridization and immunohistochemistry experiments, *in situ* hybridization was performed first. For immunohistochemistry, sections were treated with 0.2% Triton X-100 in 0.1 M PBS (PBST) for 10 min after several 0.1 M PBS washes. Then the sections were treated with 3% H_2_O_2_ and 10% methanol in PBST for 5 min. After several washes with 0.1 M PBS, sections were blocked with 3% normal goat serum (NGS) in 0.1 M PBS for 1 h. Then the diluted primary antibodies in PBST containing 1% NGS and 0.1% sodium azide were applied onto the sections overnight. The primary antibodies included rabbit anti-Ki67 (1:200, Leica Biosystems, RRID:AB_442102), mouse anti-Tuj1 (1:4,000, Promega, RRID:AB_430874), rabbit anti-Nolz-1 (Ko et al., 2013), and rabbit anti-Er81 (gift of Dr. A.C. Chang at National Yang-Ming University). On the next day, after several washes with 0.1 M PBS, sections were incubated with secondary antibodies for 1 h. For detecting Tuj1 and Er81, DTAF conjugated donkey anti-mouse (1:250, Jackson ImmunoResearch) and FITC conjugated goat-anti-rabbit (1:250, Jackson ImmunoResearch, RRID:AB_2337972) were used, respectively. For detecting Ki67 and Nolz1, biotinylated goat anti-rabbit (1:500, Vector Laboratories, RRID:AB_2313606) was used and followed by Avidin-Biotin-peroxidase complex amplification (ABC kit, Vector). The immunoreactive signals were further amplified by the TSA system.

### Quantification and Statistical Analysis

Photomicrographs of *D1R;Zswim6* or *D2R;Zswim6* double *in situ* hybridization were taken from the rostral, middle, and caudal striatum of three adult mouse brains with the aid of a confocal microscope (SP2 confocal, Leica). For each brain, confocal images were acquired from eight regions that were randomly chosen in the striatum at each anatomical level. Positive cells with good signal quality in the confocal images were counted. The total number of striatal cells that were analyzed from the three brains were as follows: Rostral level: *D1R;Zswim6*, *n* = 1,029 cells, *D2R;Zswim6*, *n* = 976 cells; Middle level: *D1R;Zswim6*, *n* = 970 cells, *D2R;Zswim6*, *n* = 984 cells; Caudal level: *D1R;Zswim6* cells, *n* = 394 cells, *D2R;Zswim6*, *n* = 590 cells. The student’s *t*-test was used for statistical analysis.

## Results

We performed *in situ* hybridization using digoxigenin-labeled riboprobes to characterize the developmental expression pattern of *Zswim6* transcripts in mouse forebrain from embryonic stages to adulthood. In parallel, we also performed *in situ* hybridization using ^35^S-isotope-labeled riboprobes in most developmental stages. We performed *in situ* hybridizations with both digoxigenin- and ^35^S-labeled riboprobes for two reasons. First, ^35^S-labeled riboprobes are presumably more sensitive than digoxigenin-labeled riboprobes in detecting low levels of *Zswim6* mRNA. Second, ^35^S-labeled riboprobes *in situ* hybridization, however, does not provide spatial resolution at the cellular level, which could be resolved by digoxigenin-labeled riboprobes *in situ* hybridization. We, therefore, performed *in situ* hybridizations with digoxigenin-labeled and ^35^S-labeled *Zswim6* riboprobes in two sets of developing brain sections. The results showed that the pattern of ^35^S-labeled riboprobes was generally in agreement with that of digoxigenin-labeled riboprobes *in situ* hybridization. These experiments with two different labeled riboprobes validated the expression pattern of *Zswim6* mRNA in the developing mouse forebrain. Detailed expression patterns of *Zswim6* mRNA in the mouse forebrain across developmental stages are summarized in Table 1.

**Table 1:** Expression pattern of *Zswim6* in the developing mouse forebrain.

Region/Age	E11.5	E12.5	E13.5	E15.5	E17.5	P0	P7	P14	Adult
Neocortex									
Cortical plate	N/A	+	++	+++	+++	+++	++	+	+
SVZ/IZ	N/A	–	–	–	–	–	–	–	–
Hippocampus	–	+	++	+++	+++	+++	+++	+++	+++
Piriform cortex	N/A	N/A	N/A	–	++	+++	+++	+++	+++
Septum	+	++	++	+	+	+/–	+/–	+/–	+/–
Olfactory bulb	N/A	N/A	N/A	++	+++	+++	+++	+++	+++
Amygdala	+	+	+	+	+	+	+	+	+
Basal Ganglia									
Striatum	N/A	N/A	+/–	+	+	+++	+++	+++	+++
LGE (striatal primordium)									
VZ	–	–	–	–	–	N/A	N/A	N/A	N/A
SVZ	+++	+++	+++	+++	+++	N/A	N/A	N/A	N/A
MZ	+/–	+/–	+	+	+	N/A	N/A	N/A	N/A
Pallidum	N/A	N/A	–	–	–	–	–	–	–
MGE (pallidal primordium)									
VZ	–	–	–	–	–	N/A	N/A	N/A	N/A
SVZ	++	++	++	+/–	+/–	N/A	N/A	N/A	N/A
MZ	++	+	–	–	–	N/A	N/A	N/A	N/A
CGE	++	++	++	+	N/A	N/A	N/A	N/A	N/A
Olfactory tubercle	N/A	N/A	N/A	–	++	+++	+++	+++	+++
Thalamus	+	+	+	++	++	++	+	+/–	–
Hypothalamus	++	++	++	+	+	+	+/–	–	–
Preoptic area	–	++	+	+	–	–	–	N/A	N/A

*The relative expression intensity of each structure is defined compared to the structure with the strongest expression at each stage. For some structures with very weak expression identified by alkaline phosphatase signals, ^35^S-radioactive data is evaluated for reference. The signal intensity is classified as: strong (+++), moderate (++), weak (+), very weak (+/−), or no expression (–). CGE, caudal ganglionic eminence; E, embryonic day; IZ, intermediate zone; LGE, lateral ganglionic eminence; MGE, medial ganglionic eminence; MZ, mantle zone; N/A, not applicable; P, postnatal day; SVZ, subventricular zone; VZ, ventricular zone*.

### *Zswim6* mRNA Expression Pattern in the Embryonic Mouse Forebrain

#### E11.5

**Figure 1:**
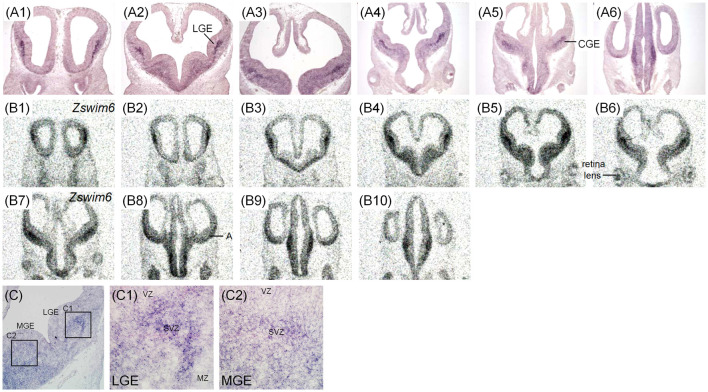
*Zswim6* mRNA expression pattern in E11.5 mouse forebrain. Expression pattern of *Zswim6* mRNA analyzed by *in situ* hybridization with digoxigenin-labeled probes **(A1–A6)** and ^35^S-labeled probes **(B1–B10)** from rostral to caudal levels. **(C)** *Zswim6* is strongly expressed in the SVZ of LGE **(C,C1)**, but is weaker in the SVZ of MGE **(C,C2)**. A, amygdala; CGE, caudal ganglionic eminence; LGE, lateral ganglionic eminence; MGE, medial ganglionic eminence; MZ, mantel zone; Sep, septum; SVZ, subventricular zone; VZ, ventricular zone.

The expression pattern of *Zswim6* mRNA in the E11.5 forebrain (*n* = 5) from rostral to caudal levels is illustrated in Figure 1. *Zswim6* mRNA was highly expressed in the subventricular zone (SVZ) of lateral ganglionic eminence (LGE, striatal anlage; Figures 1A1–A4,B1–B4,C,C1). Once the progenitor cells in the SVZ migrated into the differentiated mantle zone (MZ), *Zswim6* was down-regulated as indicated by weak signals in the MZ of LGE (Figures 1C,C1). *Zswim6* was also expressed in the SVZ and MZ of medial ganglionic eminence (MGE) and caudal ganglionic eminence (CGE; Figures 1A2–5,B3–B6,C,C2). Low levels of *Zswim6* expression were found in the ventricular zone (VZ) of the three ganglionic eminences. *Zswim6* mRNA was detected in the primordium of the amygdala (Figure 1B8) and hypothalamus (Figures 1A6,B9,B10). *Zswim6* expression was detected in the primordia of the retina and lens (Figure 1B6).

#### *Zswim6* mRNA Expression at E12.5 and E13.5

**Figure 2:**
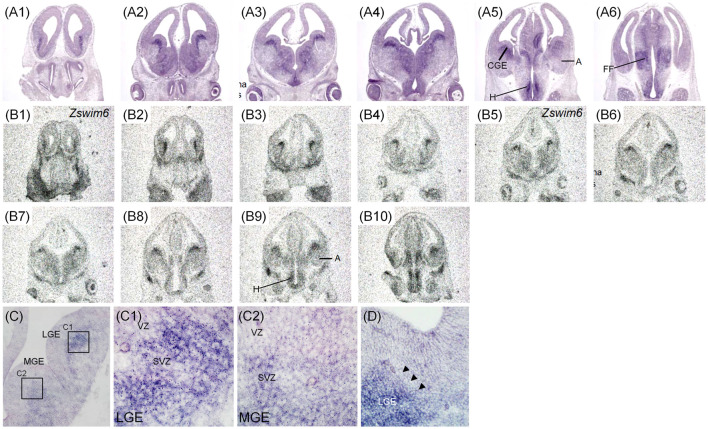
*Zswim6* mRNA expression pattern in E12.5 mouse forebrain. Expression pattern of *Zswim6* mRNA analyzed by *in situ* hybridization with digoxigenin-labeled probes **(A1–A6)** and ^35^S-labeled probes **(B1–B10)** from rostral to caudal levels. **(C)** *Zswim6* is strongly expressed in the SVZ of LGE **(C,C1)**, but is weaker in the SVZ of MGE **(C,C2)**. **(D)** *Zswim6* is highly expressed in the SVZ of LGE. There is a boundary at the dorsal-most part of the SVZ of LGE (arrowheads). A, amygdala; CGE, caudal ganglionic eminence; CP, cortical plate; H, hypothalamus; Hip, hippocampus; LGE, lateral ganglionic eminence; MGE, medial ganglionic eminence; MZ, mantel zone; SVZ, subventricular zone; VZ, ventricular zone.

**Figure 3:**
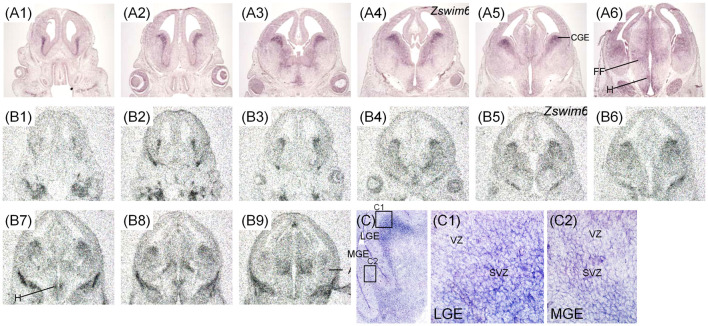
*Zswim6* mRNA expression pattern in E13.5 mouse forebrain. Expression pattern of *Zswim6* mRNA detected by *in situ* hybridization with digoxigenin-labeled probes **(A1–A6)** and ^35^S-labeled probes **(B1–B9)** from rostral to caudal levels. **(C)** *Zswim6* is strongly expressed in the SVZ of LGE **(C,C1)**. Weak expression of *Zswim6* is present in the SVZ of MGE **(C,C2)**. A, amygdala; CGE, caudal ganglionic eminence; CP, cortical plate; FF, Fields of Forel; H, hypothalamus; LGE, lateral ganglionic eminence; MGE, medial ganglionic eminence; MZ, mantel zone; SVZ, subventricular zone; VZ, ventricular zone.

The expression pattern of *Zswim6* in E12.5 (*n* = 10) and E13.5 forebrain (*n* = 7) from rostral to caudal levels are illustrated in Figures 2, 3. The expression pattern of *Zswim6* at E12.5 was similar to that at E13.5. *Zswim6* was weakly expressed in the septum (Figures 2A1,B1, 3A1,B1). *Zswim6* expression was strong in the SVZ but weak in the MZ of LGE, suggesting that *Zswim6* expression was down-regulated in progenitor cells of the LGE while undergoing neuronal differentiation (Figures 2A2,A3,B2–B5,C,C1, 3A2–A4,B2–B5,C,C1). In the MGE and CGE, *Zswim6* was also expressed in the SVZ and MZ, but not in the VZ (Figures 2A2–A5,C,C2, 3A2–A5,B2–B5,C,C2). Notably, *Zswim6* expression formed a boundary at the dorsal-most part of the SVZ of LGE at E12.5 and E13.5 (Figures 2D, 3A2,A3). At caudal levels, *Zswim6* expression was detected in the hypothalamus (Figures 2A5,A6,B9,B10, 3A5,A6,B7–9). Fields of Forel, one of the subdivisions of the hypothalamus, had the highest expression (Figures 3A6,B9). *Zswim6* was homogenously expressed in the primordia of the retina and lens (Figures 2A3,B5, 3A2,B3).

#### *Zswim6* mRNA Expression at E15.5

**Figure 4:**
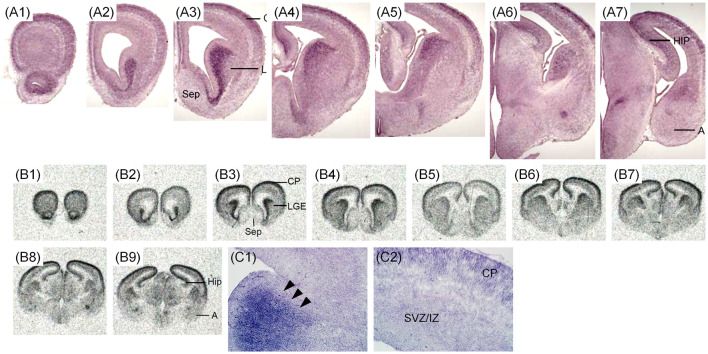
*Zswim6* mRNA expression pattern in E15.5 mouse forebrain. Expression pattern of *Zswim6* mRNA detected by *in situ* hybridization with digoxigenin-labeled probes** (A1–A7)** and ^35^S-labeled probes **(B1–B9)** from rostral to caudal levels. *Zswim6* is mainly expressed in the SVZ of developing striatum with a border at the dorsal-most part (arrowheads in **C1**). *Zswim6* is expressed in the developing cortex **(C2)**. A, amygdala; CGE, caudal ganglionic eminence; CP, cortical plate; Hip, hippocampus; IZ, intermediate zone; LGE, lateral ganglionic eminence; MGE, medial ganglionic eminence; Sep, septum; SVZ, subventricular zone; VZ, ventricular zone.

The expression pattern of *Zswim6* mRNA in the E15.5 forebrain (*n* = 8) from rostral to caudal levels are illustrated in Figure 4. *Zswim6* was strongly expressed in the early developing olfactory bulbs (OB; Figures 4A1,B1). At E15.5, strong expression of *Zswim6* was maintained in the SVZ and the expression was low in the MZ of the developing striatum (Figures 4A2–A5). As in earlier stages, *Zswim6* expression showed a boundary at the dorsal-most part of the developing striatum (Figure 4C1). *Zswim6* expression was detected in the developing cortical plates (CP) and hippocampus from rostral to caudal levels (Figures 4A2–A7,B2–B9,C2). Weak expression of *Zswim6* was detected in the thalamus and hypothalamus (Figures 4A6,A7,B8,B9).

#### *Zswim6* mRNA Expression at E17.5

**Figure 5:**
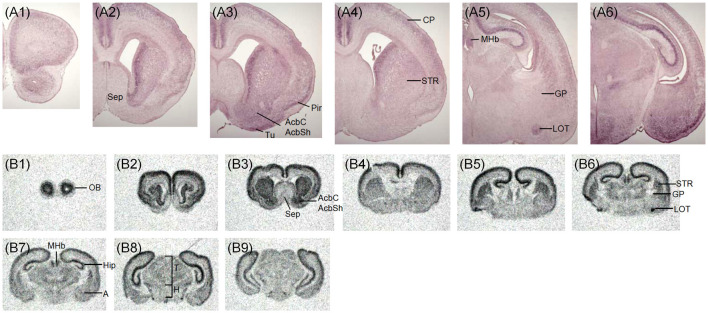
*Zswim6* mRNA expression pattern in E17.5 mouse forebrain. Expression pattern of *Zswim6* mRNA detected by *in situ* hybridization with digoxigenin-labeled probes **(A1–A6)** and ^35^S-labeled probes **(B1–B9)** from rostral to caudal levels. A, amygdala; AcbC, nucleus accumbens, core; AcbSh, nucleus accumbens, shell; CP, cortical plate; GP, globus pallidus; H, hypothalamus; HIP, hippocampus; IZ, intermediate zone; LOT, nucleus of the lateral olfactory tract; MGE, medial ganglionic eminence; MHb, medial habenular nucleus; Pd, pallidum primordium; Pir, piriform cortex; POA, preoptic area; Sep, septum; SVZ, subventricular zone; T, thalamus; Tu, olfactory tubercle.

The expression pattern of *Zswim6* mRNA in the E17.5 forebrain (*n* = 5) from rostral to caudal levels is illustrated in Figure 5. In the developing OB, striatum, and septal area, the expression pattern of *Zswim6* at E17.5 was similar to that in the E15.5 forebrain. In the developing cortex, *Zswim6* expression was detected in the orbital cortex (Figure 5A1). Dorsal to the *Zswim6*-positive piriform cortex and olfactory tubercle, *Zswim6* was expressed in the CP from rostral to caudal levels (Figures 5A2–A6,B2–B6). The cingulate cortex had strong expression (Figures 5A4,B3,B4). The expression of *Zswim6* in the striatum appeared to have a dorsal to ventral decreasing gradient at the level where anterior commissure appeared (Figure 5B4). Other structures positive for *Zswim6* expression included the medial habenular nucleus, the nucleus of the lateral olfactory tract, and the hippocampus (Figures 5A5,A6,B6,B7).

### *Zswim6* mRNA Expression in Postnatal Mouse Forebrain

#### *Zswim6* mRNA Expression at P0 and P7

**Figure 6:**
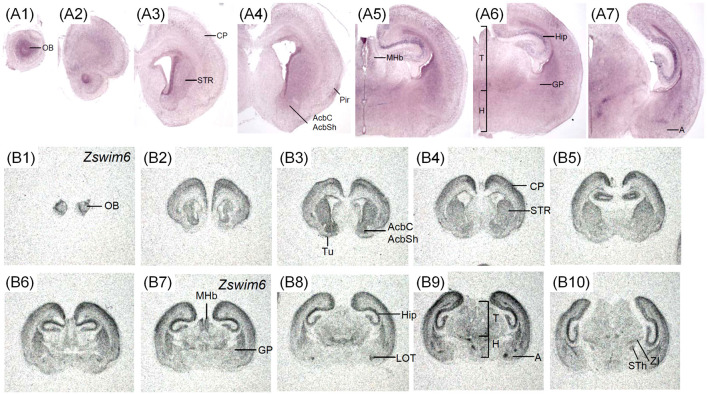
*Zswim6* mRNA expression pattern in P0 mouse forebrain. *Zswim6* mRNA signals were detected by *in situ* hybridization with digoxigenin-labeled probes **(A1–A7)** and ^35^S-labeled probes **(B1–B10)** from rostral to caudal levels at P0. Moderate to strong *Zswim6* signals were expressed in the olfactory bulb, dorsal and ventral striatum, cortex, hippocampus, thalamus, amygdala and medial habenular nucleus. A, amygdala; AcbC, nucleus accumbens, core; AcbSh, nucleus accumbens, shell; AO, anterior olfactory nucleus; Cere, cerebellum; Cg, cingulate cortex; CP, cortical plate; CTX, cortex; DG, dentate gyrus; GP, globus pallidus; GrO, granular cell layer of the olfactory bulb; H, hypothalamus; Hip, hippocampus; MHb, medial habenular nucleus; Mi, mitral cell layer of the olfactory bulb; ne, neuroepithelium; OB, olfactory bulb; Pir, piriform cortex; T, thalamus; Tu, olfactory tubercle; STh, subthalamic nucleus; STR, striatum; ZI, zona incerta.

**Figure 7:**
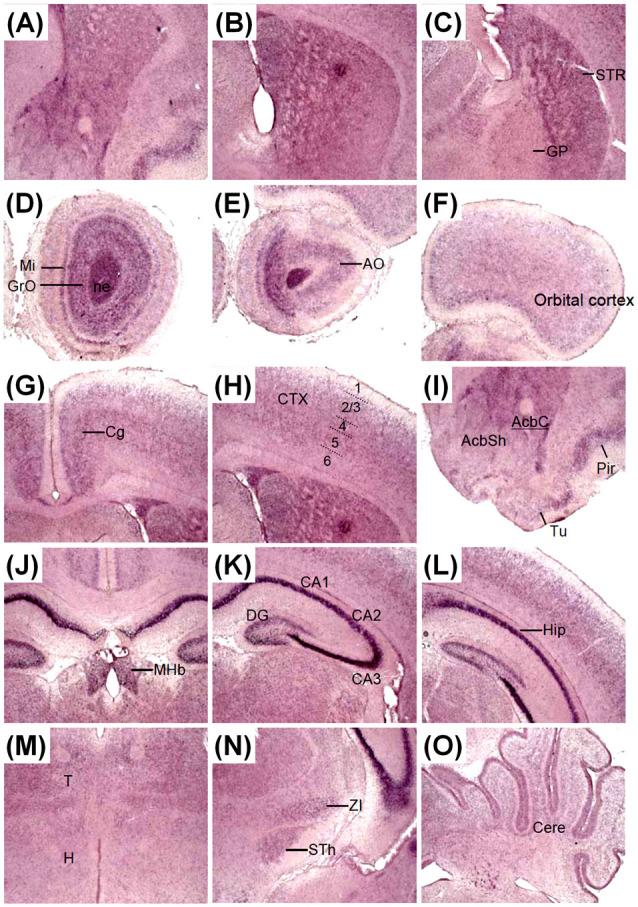
*Zswim6* mRNA expression pattern in P7 mouse forebrain. *Zswim6* mRNA signals were detected from rostral to caudal levels at P7 (**A–C**). Moderate to strong *Zswim6* signals were expressed in the olfactory bulb (**D,E**), dorsal and ventral striatum **(A–C, I)**, cortex **(F–I)**, hippocampus **(J–L)**, thalamus **(M,N)**, cerebellum **(O)**, and medial habenular nucleus **(J)**. AcbC, nucleus accumbens, core; AcbSh, nucleus accumbens, shell; AO, anterior olfactory nucleus; Cere, cerebellum; Cg, cingulate cortex; CP, cortical plate; CTX, cortex; DG, dentate gyrus; GP, globus pallidus; GrO, granular cell layer of the olfactory bulb; H, hypothalamus; Hip, hippocampus; MHb, medial habenular nucleus; Mi, mitral cell layer of the olfactory bulb; OB, olfactory bulb; Pir, piriform cortex; T, thalamus; Tu, olfactory tubercle; STh, subthalamic nucleus; STR, striatum; ZI, zona incerta.

The expression pattern of *Zswim6* on postnatal day (P) 0 and P7 forebrain was illustrated in Figures 6, 7. *Zswim6* was persistently expressed in developing OB at P0 (Figures 6A1,B1). *Zswim6* expression was detected in the mitral cell layer and granule cell layer of OB at P7 (Figure 7D). *Zswim6* was expressed in the developing cortex (Figures 6A2–A4,B2–B4, 7F–H). The cingulate cortex in the medial cortical region was positive for *Zswim6* expression (Figures 6B3,B4, 7G). *Zswim6* signals in the cortex were further expended ventrally into the piriform cortex, olfactory tubercle and if any, the islands of Calleja (Figures 6A3,A4,B3,B4, 7A,B,I). In the striatum, *Zswim6* was persistently expressed in P0 and P7 dorsal striatum as well as in the ventral striatum, including the nucleus accumbens (Acb) core (AcbC) and shell (AcbS; Figures 6A3,A4,B2–B6, 7A–C,I). The signal intensity and expressing cell number appeared to be lower in the AcbS than that in the AcbC (Figures 6B3, 7I). A dorsal to ventral decreasing gradient of *Zswim6* expression was observed at the middle level of the striatum (Figures 6A4,B4, 7B). In the hippocampus, *Zswim6* was most strongly expressed in the pyramidal cell layer, but the weak expression was present in the granular layer of the dentate gyrus (Figures 6A5–A7,B6–B10, 7J–L). Other regions expressing *Zswim6* included the medial habenular nucleus, thalamus, zona incerta, subthalamic nucleus, the nucleus of the lateral olfactory tract, and the lateral or basolateral amygdaloid nucleus (Figures 6A5–A7,B7–B10, 7K–N). *Zswim6* expression in the thalamus and hypothalamus was down-regulated at P0 and P7 (Figures 6A6,B9, 7J).

#### *Zswim6* mRNA Expression at P14 and Adulthood

**Figure 8:**
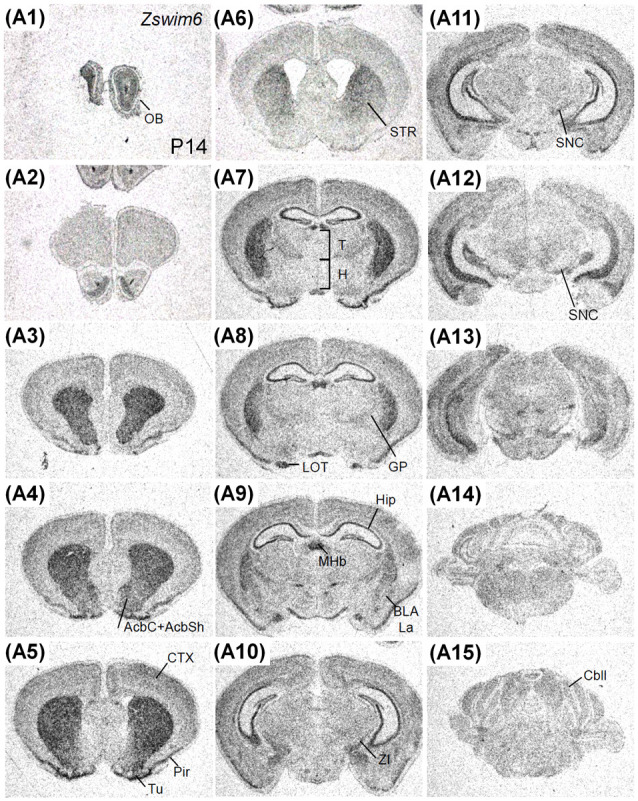
*Zswim6* mRNA expression pattern in P14 mouse forebrain. *Zswim6* mRNA signals were detected from rostral to caudal levels at P14 **(A1–A15)**. AcbC, nucleus accumbens, core; AcbSh, nucleus accumbens, shell; BLA, basolateral amygdala; Cbll, cerebellum; CTX, cortex; GP, globus pallidus; H, hypothalamus; Hip, hippocampus; La, lateral amygdaloid nucleus; LOT, nucleus of the lateral olfactory tract; MHb, medial habenular nucleus; OB, olfactory bulb; Pir, piriform cortex; SNC, substantia nigra; STR, striatum; T, thalamus; Tu, olfactory tubercle; ZI, zona incerta.

**Figure 9:**
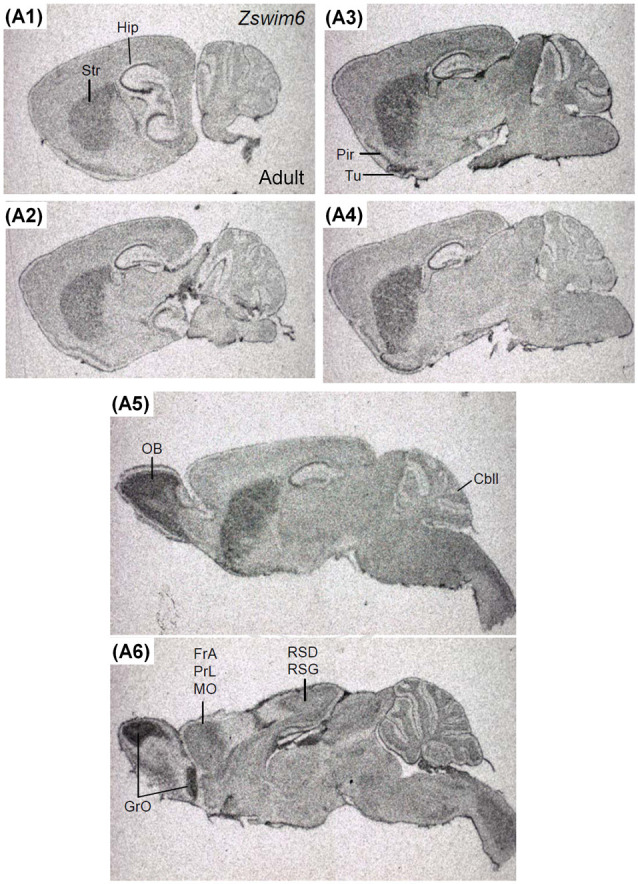
*Zswim6* mRNA expression pattern in adult mouse brain. *Zswim6* mRNA signals were detected from lateral to medial levels in parasagittal sections of the adult brain **(A1–A6)**. Cbll, cerebellum; FrA, frontal association cortex; GrO, granular cell layer of the olfactory bulb; Hip, hippocampus; MO, medial orbital cortex; OB, olfactory bulb; Pir, piriform cortex; PrL, prelimbic cortex; RSD, retrosplenial dysgranular cortex; RSG, retrosplenial granular cortex; Str, striatum; Tu, olfactory tubercle.

The expression pattern of *Zswim6* in the P14 forebrain (*n* = 3; Figure 8) was similar to that in the P7 forebrain. However, the expression levels of *Zswim6* in the cerebral cortex and the thalamus appeared to be down-regulated at P14 compared to earlier stages (Figure 8A7). In the adult forebrain, persistent expression of *Zswim6* was detected in the OB, striatum, AcbC, AcbS, pyramidal cell layer of the hippocampus, granule cell layer of the dentate gyrus, olfactory tubercle, piriform cortex, medial habenular nucleus, and the substantia nigra (Figure 8). In the adult brain, a lateral-to-medial increasing gradient of *Zswim6* in the striatum was observed in parasagittal sections (Figures 9A1–A6). *Zswim6* expression in the cortex was lower than that in the striatum in adulthood (Figures 9A1–A6).

### Cell-Type Analysis of *Zswim6* Expression

#### *Zswim6* Is Expressed in Nolz1-Positive but Er81-Negative Differentiating Progenitors of LGE

**Figure 10:**
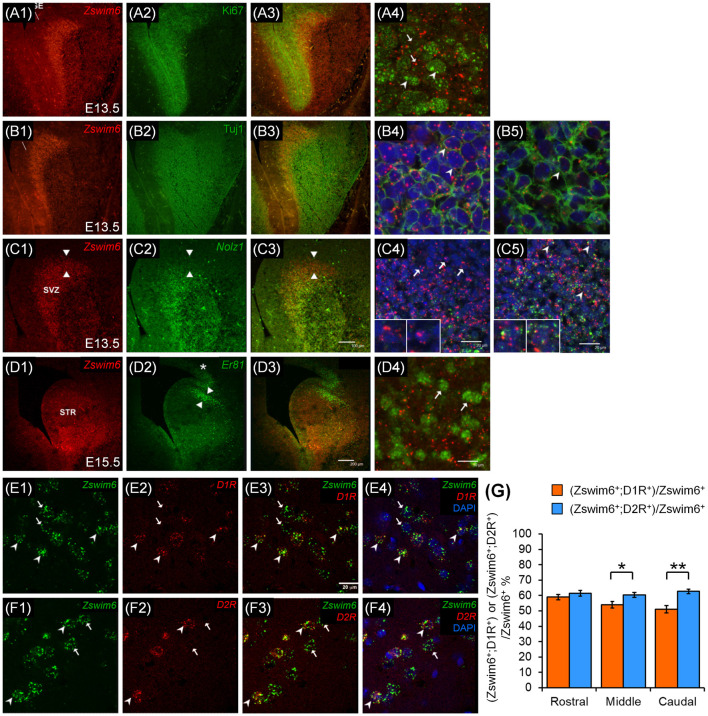
*Zswim6* are expressed in Nolz1-positive neurons in the LGE at E13.5 and striatonigral and striatopallidal neurons in adult brains. *Zswim6* is mainly expressed in the SVZ of the LGE at E13.5 **(A1,B1)**, whereas Ki67-positive **(A2)** and Tuj1-positive **(B2)** cells are located in the VZ and the MZ of the LGE, respectively. In the overlapping zone **(A3)**, *Zswim6*-positive cells express none or at most low levels of Ki67 (arrows, **A4**). Cell expressing high levels of Ki67 contained none or low levels of *Zswim6* (arrowheads, **A4**). Colocalization of Tuj1 protein and *Zswim6* mRNA **(B3)** is detected in cells of SVZ **(B4)** and MZ **(B5)**. **(C,D)** Both *Zswim6* **(C1)** and *Nolz-1* **(C2)** are expressed in the SVZ of LGE, and their expression domains are mostly overlapped in the ventral part of SVZ **(C3)**. In the dorsal non-overlapping zone, cells expressing *Zswim6* without *Nolz-1* are present (**C3**, the area between arrowheads; **C4**, insets show high magnification of the cells indicated by arrows). In the *Zswim6* and *Nolz-1* overlapped regions, *Zswim6* is colocalized with *Nolz-1* (**C5**, insets show high magnification of cells indicated by arrowheads). At E15.5, *Zswim6* mRNA **(D1)** is expressed in the SVZ of the striatal primordium, while Er81 marks the dorsal-most part of the striatal primordium (**D2**, between arrowheads). Although *Zswim6* expression is overlapped with Er81 at the dorsal-most striatum **(D3)**, at the single-cell level, *Zswim6* mRNA signals (**D4**, arrows) are not co-localized with Er81-positive cells. **(E,F)** Confocal images of double *in situ* hybridization show that some *Zswim6*-positive cells (**E1,E3,E4**; arrowheads) were co-localized with *D1R* **(E2–E4)** in the mature striatum, whereas others express *Zswim6* only (**E1,E3,E4**; arrows). In the double *in situ* hybridization of *Zswim6* and *D2R*, some *Zswim6*-positive cells (**F1,F3,F4**; arrowheads) were co-localized with *D2R* **(F2–F4)** in the mature striatum, while others expressed *Zswim6* only (**F1,F3,F4**; arrows). **(G)** Quantification of the co-localization ratios of *Zswim6* and *D1R* or *D2R* in the mature striatum from the rostral to caudal levels. *p < 0.05, **p < 0.001, *n* = 3, student’s *t*-test. LGE, lateral ganglionic eminence; MZ, mantle zone; STR, striatum; SVZ, subventricular zone.

To determine whether *Zswim6* was expressed in the population of proliferating or differentiating progenitors, *Zswim6* mRNA was double-labeled with the proliferating marker of Ki67 or the differentiating marker of Tuj1 in the E13.5 forebrain (Figures 10A1–A4,B1–B5). In the SVZ of LGE, *Zswim6*-positive cells express none or at most low levels of Ki67, and cells expressing high levels of Ki67 contained none or low levels of *Zswim6* (Figures 10A1–A4). By contrast, Tuj1-positive cells were found to co-express *Zswim6* in the SVZ of LGE (Figures 10B1–B4). Moreover, in the differentiated MZ of LGE, which contained high levels of Tuj1 but low levels of *Zswim6*, Tuj1 was also colocalized with *Zswim6* (Figure 10B5). These findings indicated that *Zswim6*-positive cells in the SVZ of LGE were postmitotic cells undergoing early stages of neuronal differentiation in the developing striatum.

*Nolz-1* is a marker of differentiating progenitors of striatal projection neurons (Chang et al., 2004). Similar to *Zswim6*, *Nolz-1* is also highly expressed in the SVZ of E13.5 LGE. We determined whether *Zswim6* and *Nolz-1* were expressed in the same progenitor population. The double *in situ* hybridization showed that *Zswim6* and *Nolz-1* mRNA expression domains overlapped in the SVZ of LGE except the dorsal-most part of LGE where *Nolz-1* expression was absent (Figures 10C1–C3). At the single-cell level, co-localization of *Zswim6* and *Nolz1* was found in the progenitors of SVZ (Figure 10C5), but not in the progenitors of the dorsal-most region of LGE (Figure 10C4). These findings indicated that, except for cells in the dorsal part of LGE, *Zswim6* and *Nolz-1* were co-expressed by the majority of early differentiating progenitor cells of the ventral LGE.

Because *Zswim6* expression showed a clear boundary at the dorsal-most part of the SVZ of LGE, we further examined whether *Zswim6* was co-localized with Er81, a marker of dorsal LGE (Stenman et al., 2003). Cells expressing Er81 did not co-express *Zswim6* in the dorsal LGE at E15.5 (Figures 10D1–D4).

#### *Zswim6* Is Expressed in Both Striatonigral and Striatopallidal Neurons

Because *Zswim6* was expressed in the adult striatum, we investigated whether *Zswim6* was specifically expressed in the populations of dopamine D1 receptor (D1R)-expressing striatonigral neurons and/or dopamine D2 (D2R)-expressing striatopallidal neurons. The double *in situ* hybridization of *Zswim6* and *D1R* or *D2R* showed co-expression of *Zswim6* and *D1R* (Figures 10E1–E4) or *D2R* (Figures 10F1–F4). Quantitative analysis indicated that the co-localization ratio of *D2R^+^;*Zswim6^+^ cells/*Zswim6^+^ cells* was moderately higher than that of *D1R^+^;*Zswim6^+^ cells/*Zswim6^+^* cells (Figure 10G). At the rostral level, the co-expression ratios of *D1R^+^;Zswim6^+^*/*Zswim6^+^* cells and *D2R^+^;*Zswim6^+^/*Zswim6^+^* cells were 58.99% ± 1.72 and 61.46% ± 1.78%, respectively (*p* = 0.3134, *n* = 3). At the middle level, the co-expression ratios of *D1R^+^;*Zswim6^+^/*Zswim6^+^* cells and *D2R^+^;Zswim6^+^*/*Zswim6^+^* cells were 53.99% ± 2.07% and 60.29% ± 1.56%, respectively (*p* = 0.0163, *n* = 3). At the caudal level, where the anterior commissure crosses the midline, the co-expression ratios of *D1R^+^;Zswim6^+^*/*Zswim6^+^* cells and *D2R^+^;Zswim6^+^*/*Zswim6^+^* cells were 50.98% ± 2.42% and 62.73% ± 1.46%, respectively (*p* = 0.0003, *n* = 3). In brief, relative to the ratio of *D1R^+^;*Zswim6^+^ cells, the co-localization ratios of *D2R^+^;*Zswim6^+^ cells were 104.19% ± 3.01% at the rostral level (*p* = 0.3134, *n* = 3), 111.67% ± 2.89% at the middle level (*p* = 0.0163, *n* = 3) and 123.04% ± 2.86% at the caudal level (*p* = 0.0003, *n* = 3). Therefore, *Zswim6*-positive neurons were moderately enriched in striatopallidal neurons than striatonigral neurons in the middle and caudal striatum at adulthood.

## Discussion

In the present study, we have comprehensively characterized the expression pattern of *Zswim6* mRNA in the developing mouse forebrain. In the early forebrain development from E11.5 to E13.5, *Zswim6* mRNA was highly expressed in the SVZ of LGE (striatal anlage). At E15.5 and E17.5, *Zswim6* expression was detected not only in the developing striatum but also in other brain regions, including the developing cerebral cortex, hippocampus, medial habenular nucleus, and olfactory bulb. *Zswim6* expression was persistently expressed in the postnatal brain, though the expression levels appeared to be down-regulated in the striatum and cortex.

Consistent with the previous study (Tischfield et al., 2017), *Zswim6* was prominently expressed in the SVZ, but not VZ, of developing striatum. The SVZ is a transition zone from cell proliferation to differentiation. We found that most cells expressing high levels of the proliferating marker Ki67 contained none or at most low levels of Zswim6 in the SVZ. *Zswim6* was co-expressed with the differentiating marker Tuj1 in the SVZ and MZ, indicating that *Zswim6* is expressed in postmitotic differentiating neurons. Moreover, *Zswim6* was colocalized with *Nolz-1*, a marker of early differentiating progenitors in the SVZ of LGE (Chang et al., 2004; Ko et al., 2013; Chen et al., 2020), suggesting that *Zswim6* is expressed in differentiating striatal progenitors. We further found that *Zswim6* mRNA was colocalized with *D1R*-expressing striatonigral and *D2R*-expressing striatopallidal neurons of the adult striatum. Collectively, these findings indicate that *Zswim6* is expressed in early differentiating progenitors, and it is persistently expressed in striatal projection neurons of the adult brain.

The SVZ of LGE contains two neuronal progenitor populations. *Dlx*^+^;*Isl1*^+^ cells make up the majority of progenitors in the ventral part of LGE that gives rise to striatal projection neurons, whereas *Dlx*^+^;*Er81*^+^ cells in the dorsal-most part of LGE comprises progenitors of olfactory bulb interneurons (Stenman et al., 2003). *Zswim6* mRNA was mostly expressed in the ventral LGE. Double labeling of *Zswim6* and *Nolz-1*, a marker of differentiating striatal projection neurons (Chang et al., 2004; Ko et al., 2013; Chen et al., 2020) showed that *Zswim6* and *Nolz-1* were colocalized in the progenitors of ventral LGE. Because *Nolz-1* is co-expressed with *Isl1* in early differentiating striatal projection neurons (Chang et al., 2004), and *Zswim6* is co-localized with *Nolz-1*, *Zswim6* is thus presumably expressed in *Dlx*^+^;*Isl1*^+^ progenitors of ventral LGE that gives rise to striatal projection neurons. In contrast to the high level in the ventral LGE, a low level of *Zswim6* mRNA was present in the dorsal LGE in which *Zswim6* was not colocalized with Er81. Therefore, *Zswim6* is not expressed in *Dlx*^+^;*Er81*^+^ progenitors of dorsal LGE where olfactory bulb interneurons reside.

Previous studies have identified several transcriptional regulators that are enriched in the LGE, MGE and CGE, e.g., Dlx1, Dlx2, Isl1, RARβ, Nolz-1, and SP9, and these transcriptional factors regulate cell-type specification, migration, and differentiation (Liao et al., 2008; Rubenstein and Campbell, 2013; Tao et al., 2019; Chen et al., 2020). *Zswim6* is expressed in the LGE, MGE, and CGE and *Zswim6* is up-regulated at the late progenitor state of the maturation trajectory of CGE at E14.5 (Mayer et al., 2018). The developmental expression pattern of *Zswim6* suggests that Zswim6 may be involved in the neurogenesis of striatal neurons. Tischfield et al. (2017) have shown that genetic deletion of *Zswim6* resulted in a moderate loss of striatal neurons but marked reductions in dendritic complexity and length, and dendritic spines of striatal neurons. No evident decreases in cell proliferation and increases in cell apoptosis were observed in the embryonic striatum of *Zswim6* knockout mice (Tischfield et al., 2017). Moreover, they found no changes in the expression of several striatonigral and striatopallidal-enriched genes in *Zswim6* knockout brains. Interestingly, the knockout mice are hyperactive in locomotion and they exhibit deficits in rotarod learning and repetitive motor behaviors. Notably, our present study has shown that *Zswim6* is expressed at a higher level in *D2R*-expressing striatopallidal neurons than *D1R*-expressing striatonigral neurons, which is in agreement with the previous FACS-array study (Lobo et al., 2006), suggesting that *Zswim6* may regulate striatal dopamine neurotransmission. Although *D1R* and *D2R* mRNA levels were not altered in the striatum of *Zswim6* knockout mice, the knockout mice exhibited enhanced locomotion in response to dopamine agonist amphetamine (Tischfield et al., 2017). The striatum has been proposed as a key region of dopamine neurotransmission that is involved in the pathophysiology of schizophrenia (McCutcheon et al., 2019). Given that *Zswim6* is associated with schizophrenia, it is of interest to investigate whether abnormal striatal dopamine neurotransmission occurs in *Zswim6* knockout mice that may contribute to schizophrenia-like phenotypes.

Strong *Zswim6* expression is detected in the CA1 and CA3 of the hippocampus. The hippocampus is known to be involved in the pathogenesis of schizophrenia (Ho et al., 2017; Roeske et al., 2020). Interestingly, *Zswim6* is expressed in the medial habenula in which the schizophrenia-risk gene *ErbB4* is also highly expressed (Steiner et al., 1999). Although the pathological role of the habenular nucleus is less characterized in schizophrenia, the habenular complex is a hub region for regulating motivation and emotion that are affected in schizophrenia (Sandyk, 1991; Namboodiri et al., 2016; Zhang et al., 2017).

Increasing evidence suggests that poorly annotated non-coding variants contribute to the pathophysiology of schizophrenia through unknown mechanisms (Takata, 2019). As non-coding regions can regulate transcription and translation of protein-coding regions, non-coding variants may cause dysregulation of protein-coding genes. Previous studies have identified overlapping mutations in different neuropsychiatric disorders, suggesting that alterations of common special regulatory elements may underlie some neuropsychiatric disorders (Hoischen et al., 2014; Li et al., 2016; Vissers et al., 2016). Bioinformatic analysis of de novo mutations from the NPdenova database has found biological pathways and non-coding regulatory elements of candidate genes that are shared among schizophrenia, autistic spectrum disorders, intellectual disability, and encephalopathy (Li et al., 2016). It would be of interest to identify non-coding variants of *Zswim6* and study the non-coding regions that may regulate *Zswim6* expression, which may help understand the pathology of *Zswim6*-associated neurological disorders.

Our previous study has characterized the expression pattern of *Zswim5*, another member of the *Zswim* family, in the developing mouse forebrain (Chang et al., 2020). In contrast to the enriched expression of *Zswim6* in the LGE, *Zswim5* is preferentially expressed in the adjacent structure of MGE in the ventral forebrain. ZSWIM5 is expressed in progenitors of GABAergic interneurons that tangentially migrate from the MGE to the developing cortex. Notably, in addition to the high level of expression in the LGE, *Zswim6* mRNA is also expressed in the MGE as early as E11.5, suggesting that *Zswim6* may also be expressed in progenitors of cortical interneurons. *Zswim6* signals were detected in the developing cortex. However, it remains to be clarified whether *Zswim6* is expressed in interneurons and/or projection neurons of the developing cortex. The cell type characterization of *Zswim6* expression will help explore the neuronal function of *Zswim6* and the pathology of *Zswim6*-associated neurological diseases.

## Data Availability Statement

The original contributions presented in the study are included in the article, further inquiries can be directed to the corresponding author/s.

## Ethics Statement

The animal study was reviewed and approved by Institutional Animal Care and Use Committee at National Yang-Ming University.

## Author Contributions

C-CC, H-YK, S-YC, W-TL, and K-ML performed experiments. TS provided reagents. C-CC, H-YK, and F-CL analyzed data and wrote the manuscript. All authors contributed to the article and approved the submitted version.

## Conflict of Interest

The authors declare that the research was conducted in the absence of any commercial or financial relationships that could be construed as a potential conflict of interest.
